# Prostaglandin E2-EP1 and EP2 receptor signaling promotes apical junctional complex disassembly of Caco-2 human colorectal cancer cells

**DOI:** 10.1186/1471-2121-9-63

**Published:** 2008-12-02

**Authors:** Marcelo N Tanaka, Bruno L Diaz, Wanderley de Souza, Jose A Morgado-Diaz

**Affiliations:** 1 Divisão de Biologia Celular, Coordenação de Pesquisa, Instituto Nacional de Câncer, Rua André Cavalcanti 37, 5° Andar, Rio de Janeiro, RJ, CEP: 20230-051, Brazil; 2 Laboratório Intermediário de Inflamação, Instituto de Biofísica Carlos Chagas Filho, Universidade Federal do Rio de Janeiro, Ilha do Fundão, 21941-902, Rio de Janeiro, RJ, Brazil; 3 Laboratório de Ultraestrutura Celular Hertha Meyer, Instituto de Biofísica Carlos Chagas Filho, Universidade Federal do Rio de Janeiro, Ilha do Fundão, 21941-902, Rio de Janeiro, RJ, Brazil

## Abstract

**Background:**

The apical junctional complex (AJC) is a dynamic structure responsible to maintain epithelial cell-cell adhesions and it plays important functions such as, polarity, mechanical integrity, and cell signaling. Alteration of this complex during pathological events leads to an impaired epithelial barrier by perturbation of the cell-cell adhesion system. Although clinical and experimental data indicate that prostaglandin E_2 _(PGE_2_) plays a critical function in promoting cell motility and cancer progression, little is known concerning its role in AJC disassembly, an event that takes place at the beginning of colorectal tumorigenesis. Using Caco-2 cells, a cell line derived from human colorectal cancer, we investigated the effects of prostaglandin E_2 _(PGE_2_) treatment on AJC assembly and function.

**Results:**

Exposition of Caco-2 cells to PGE_2 _promoted differential alteration of AJC protein distribution, as evidenced by immunofluorescence and immunoblotting analysis and impairs the barrier function, as seen by a decrease in the transepithelial electric resistance and an increase in the permeability to ruthenium red marker. We demonstrated the involvement of EP1 and EP2 prostaglandin E_2 _receptor subtypes in the modulation of the AJC disassembly caused by prostanoid. Furthermore, pharmacological inhibition of protein kinase-C, but not PKA and p38MAPK significantly prevented the PGE_2 _effects on the AJC disassembly.

**Conclusion:**

Our findings strongly suggest a central role of Prostaglandin E2-EP1 and EP2 receptor signaling to mediate AJC disassembly through a mechanism that involves PKC and claudin-1 as important target for the TJ-related effects in human colorectal cancer cells (Caco-2).

## Background

Tight junctions (TJs) and the subjacent adherens junctions (AJs) constitute the apical junctional complex (AJC), which is responsible to maintain the epithelial phenotype [1,2]. TJs form a semi-permeable diffusion barrier in an ion- and size- selective manner through the paracellular pathway and have a fence function to maintain cell polarity as a boundary between the apical and basolateral plasma membrane domains [3]. AJs are the main adhesive junctions involved in the mechanical strength of tissues [4]. Recent studies suggest that these complexes not only mediate cell-cell adhesion, but are also engaged in signal transduction [5]. E-cadherin, the main protein of AJs interacts with the cytoskeleton via association with cytoplasmic proteins, the α-, β – and p120-catenins. Whereas β-catenin associated with E-cadherin at the plasma membrane regulates cell-cell adhesion, cytoplasmic β-catenin is involved in signal transduction and activation of genes, which play important roles in the development and progression of colorectal carcinoma [6]. The role of TJ proteins is less understood in this context. A number of integral membrane proteins associated with TJs have been identified during recent years. These include occludin, junctional adhesion molecule (JAM) and the claudin family consisting of at least 24 members. PDZ proteins of the MAGUK family are other integrant proteins of TJs, which are localized at the membrane-cytoskeleton interfaces of cell-cell contacts. They include the zonula occludens proteins ZO-1, ZO-2 and ZO-3, which are potentially involved in cell signaling [7,8]. The role of ZO-1 protein is related to the interaction with the transcriptional factor ZONAB, known to regulate many events such as growth and proliferation [9].

Prostaglandins (PGs) are bioactive lipid molecules produced by the cyclooxygenase enzymes COX-1 and COX-2, and exert diverse physiological actions in the gastrointestinal tract including maintenance of mucosal integrity, regulation of secretion and cell motility [10]. Clinical and experimental data indicate that prostaglandin E_2 _(PGE_2_) plays a predominant role in promoting cancer progression. It was reported that PGE_2 _stimulates EP receptor signaling with subsequent enhancement of cellular proliferation, promotion of angiogenesis, inhibition of apoptosis, stimulation of invasion/motility of colon cancer cells, as well as tumorigenic potential in intestinal epithelial cells [11,12]. It has been reported that both COX-2 and the epidermal growth factor receptor (EGFR) are activated in most human cancers. The observation that forced expression of COX-2 in human colorectal cancer (CRC) cells stimulates proliferation through EGFR activation, suggests the likelihood of a cross talk between these two pathways [13,14]. In a previous study we have demonstrated a link between the PKC, EGFR and MAPK pathways to modulate the loss of E-cadherin dependent cell-cell adhesion in Caco-2 cell [15].

PGE_2 _has also been implicated in direct EGFR activation through intracellular phosphorylation of receptor tyrosine kinase or extracellular release of a membrane-bound EGFR ligand, such as heparin-binding EGF in human colorectal cancer cells [16]. However, the involvement of EP receptor subtypes in these studies has been not reported. Furthermore, it was shown in LS174T, a human colorectal cancer cell line, that PGE_2 _induces expression of amphiregulin, an EGFR ligand, through a Protein Kinase A (PKA)-dependent mechanism [11]. Although it is known that PGE_2 _is the ligand to four EP receptors subtypes called EP1, EP2, EP3 and EP4, which are the products of separate genes [17,18], the lack of information concerning the role that each EP receptor plays hinders the understanding of PGE_2_-mediated gastrointestinal physiology alterations. Moreover, the precise role of each EP in the malignant behavior remains to be defined. Some studies have reported the participation of the EP1 and EP4 receptor in promoting tumorigenic behavior in colon carcinogenesis [12,19,20] and downregulation of subtype EP3 during colon cancer development [21]. However, the identification of EP modulating epithelial barrier function through mediation of AJC disassembly events has not been reported.

The aim of the present study was to investigate the response of Caco-2 cells to treatment with PGE_2_. We hypothesized that PGE2 would impair the AJC assembly and function of Caco-2 cells. We examined AJC protein distribution, paracellular permeability and identified the involvement of EP receptors as well as cell signaling pathways in response to prostanoid treatment. We report in this study that treatment with PGE_2 _caused a transient AJC disassembly through a network involving EP1 and EP2 receptors and PKC signaling with claudin-1 as target related to TJs effects in the human colon cancer cells, Caco-2.

## Results

### Prostaglandin E2 treatment causes a differential redistribution of the AJC proteins

**Figure 1:**
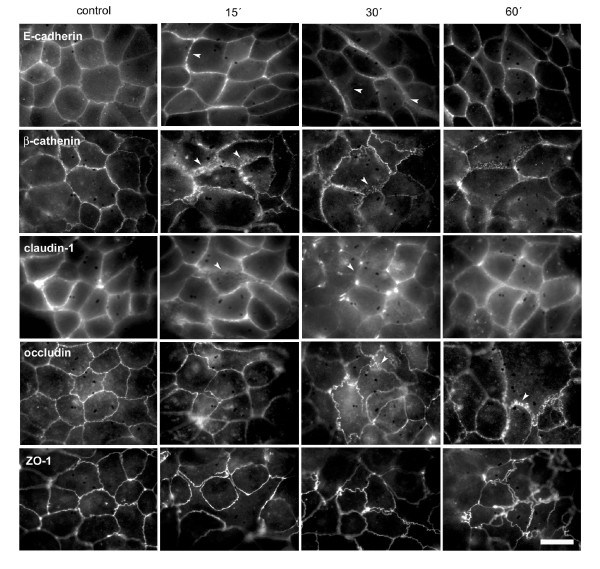
**Prostaglandin E2 treatment causes a differential redistribution of the AJC proteins**. Caco-2 cells were grown on sterile glass coverslips and after PGE_2 _treatments they were processed for immunofluorescence analysis using specific antibodies of AJC proteins, as indicated. Images show control (0 min) and treated for 15, 30 and 60 min treatments with 1 μM PGE_2_. Note that – with the exception of ZO-1 – E-cadherin, β-catenin, claudin-1 and occludin showed alterations of the staining pattern at 15 and 30 min of treatment. An apparent recovery of the labeling was observed at 60 min for E-cadherin, β-catenin and claudin-1, but not for occludin that showed membranous projections to the cytoplasm. Bar: 10 μm

Initially, we analyzed the distribution of the AJC proteins after treatment with PGE_2 _by immunofluorescence microscopy using antibodies against E-cadherin, β-catenin, claudin-1, occludin and ZO-1. Figure 1 shows a continuous and intense labeling at the cell-cell contact region for all proteins used in non-treated cells. After PGE_2 _treatment, it was possible to observe alterations in the immunostaining pattern of AJC proteins with exception of ZO-1 that remained at the membrane. After 15 min of treatment E-cadherin appears in a discontinuous and irregular string-of beads-shape at the cell-cell contacts. At 30 min internalization into the cytoplasm was observed but at 60 min there was a significant recovery of the labeling pattern. β-catenin at 15 min also showed a discontinuous and irregular labeling at the membrane with projections to the cytoplasm; at 30 min and 60 min of treatment it appears with minor translocation into the cytoplasm, however a considerable amount of the protein was still at the membrane. Immunostaining of claudin-1 at 15 min showed a discontinuous membranous staining in same regions of cell-cell contact and at 30 min this effect was more evident with strong points of labeling and a weak or absent staining in the cell-cell contact area. At 60 min there was a labeling recovery at the cell-cell contacts. Occludin appears with no alterations at 15 min, however after 30 and 60 min of treatment projections in the direction of the cytoplasm mainly at 60 min, were observed.

**Figure 2:**
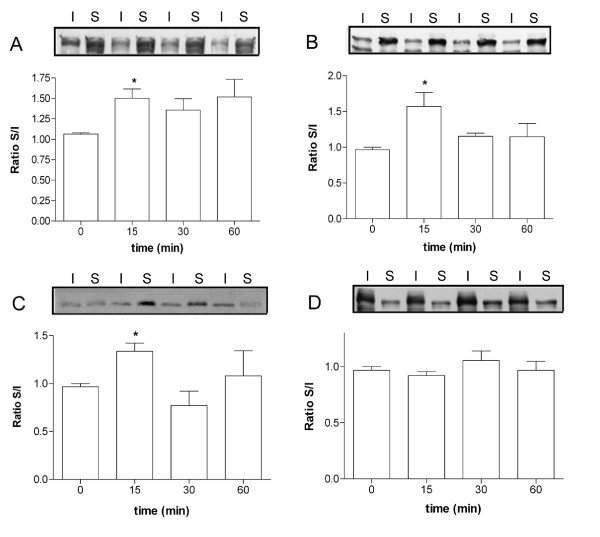
**PGE_2 _treatment alters the TX-100 solubility of AJC proteins in Caco-2 cells**. Representative immunoblots and densitometric analysis of E-cadherin (A), Beta-catenin (B), claudin-1 (C) and occludin (D) of insoluble (I) and soluble (S) fractions in Triton X-100 of cells that were untreated (0 min) or treated for 15, 30 and 60 min with 1 μM PGE2. In each case the score was calculated using the following equation: Arbitrary score= (amount of the protein in the soluble fraction)/(amount of the protein in the insoluble fraction). The score for untreated cells (0 min) was normalized as 1 in each case. Average scores S.E.M of three independent experiments are shown. Significantly different: * (P < 0.05).

We further analyzed the subcellular distribution of AJC proteins by immunoblotting using soluble and insoluble TX-100 fractions after PGE_2 _treatment (Figure 2). The distribution pattern and densitometry analysis of the AJ proteins, β-catenin, and E-cadherin showed a significant translocation from the insoluble fraction to the soluble in cells that were treated for 15 min with PGE_2 _(Figures 2A and 2B, respectively). In a similar manner, this same effect was observed for the TJ proteins, claudin-1 but not for occludin (Figures 2C and 2D). Together these results indicate that PGE_2 _treatment caused a differential redistribution of the AJC proteins.

### PGE_2 _induce ultrastructural AJC alterations with concomitant loss of TJ functionality

**Figure 3:**
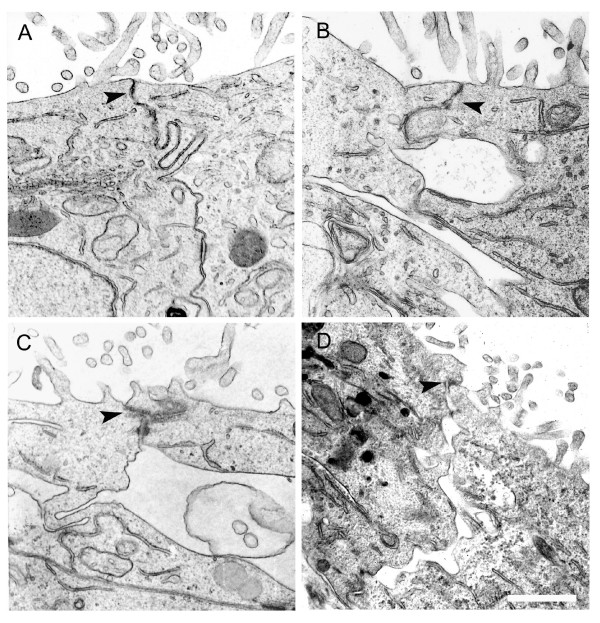
**PGE_2 _treatment influences the AJC ultrastructural characteristics of Caco-2 cells**. Caco-2 cells were grown on Transwell filters until they achieved confluence, treated with 1 μM PGE_2 _and processed for electron microscopy analysis. Representative images of thin sections of control cells (A) shows an intact AJC between two neighbor cells, however cells treated with PGE_2 _for 15 (B), 30 (C) and 60 (D) show alterations in the AJC region, mainly at the adherent junction area. Note that the TJ (Arrowheads) apparently remain unaltered. Bar: 2.5 μm. TJs: Tight Junctions.

We examined morphological alterations of AJC caused by PGE_2 _treatment using transmission electron microscopy (Figure 3). Non-treated Caco-2 cells form a well-organized monolayer with a typical junctional complex in the apical region and exhibit numerous microvilli (Figure 3A). When cells were exposed to 1 μM of PGE_2_, wide spaces at the sub apical cell-cell contact region were visible after 15 and 30 min and at 60 min there was an apparent recovery of the AJC. Although alterations well pronounced at the AJ area was observed, the TJ region apparently remained intact during the PGE_2 _treatment (Figures 3B, C and 3D).

**Figure 4:**
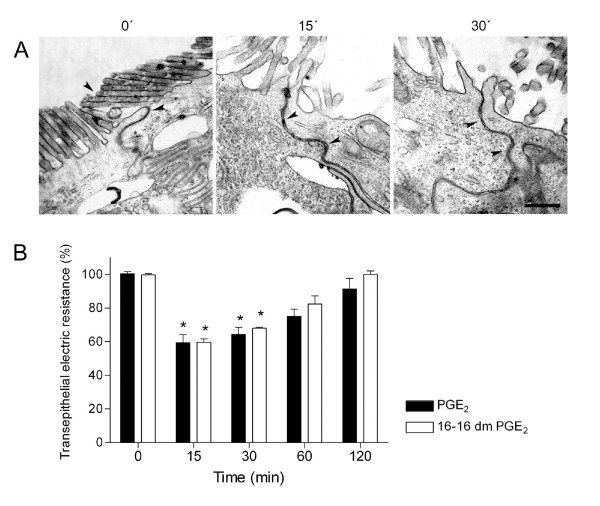
**PGE_2 _affect the paracellular permeability in Caco-2 cells**. Cells were cultured on Transwell polycarbonate filters and the TJ functionality was analyzed by the ruthenium red technique (A) and by measuring the Transepithelial electrical resistance (TER) (B). A: Representative images of thin sections of control cells showing the ruthenium red in the apical region in control cells and in cells treated with PGE_2_, as indicated. Cells incubated with the prostanoid revealed extensive spaces in the junctional complex area and permeation of the marker between the intercellular spaces. Bar: 0.8 μm. *Arrowheads*: ruthenium red. B. TER was measured in different conditions as indicated. Observe that PGE_2 _and its analogue, PGE_2 _16-16 dm PGE_2_, caused a significant drop of the TER (*P < 0.01 compared with untreated cells). The effect was visible at 15 and 30 min, however at 60 and 120 min a recovery of the TER was observed.

In order to verify TJ functionality after PGE_2 _treatment, the epithelial barrier function was assessed in individual cell junctions, using the ruthenium red technique and electron microscopy and in the cell monolayers by monitoring the TER. As seen in Figure 4, ruthenium red added to the apical region did not permeate through the TJs of untreated cells, but it permeated through the paracellular space in cells treated with PGE_2_, both at 15 and 30 min. Next, the permeability to ions of confluent Caco-2 cells was assessed by TER measurements, which showed a value of about 400 Ω.cm^2 ^(100%) in untreated cells. However, PGE_2 _treatment caused a significant drop of the TER (41% and 36%) after 15 and 30 min, respectively, but after 60 and 120 min there was a recovery. Additionally, using 16, 16-dimethyl Prostaglandin E_2 _(16, 16-dm PGE_2_), a synthetic analogue of PGE_2_, we confirmed similar effects to those observed when cells were treated with PGE_2 _(Figure 4B).

### Identification of Prostaglandin E_2 _Receptor Subtypes EP involved in the AJC disassembly

**Figure 5:**
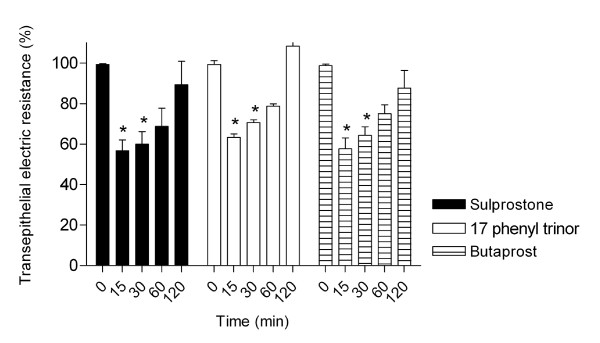
**Effects of EP agonists on the transepithelial electric resistance**. Caco-2 cells were grown to confluence on Transwell polycarbonate filters and the TER was measured before and after treatment with agonists. All the agonists caused a drop of the TER at 15 and 30 min and at 60 and 120 min a recovery was observed. S.E.M of three independent experiments are shown. Significantly different: * (P < 0.05).

PGE_2 _is known to interact with four different types of cell surface prostaglandin E receptors (EP1, EP2, EP3 and EP4), which in turn activate different signaling pathways [10]. In the present study we identified PGE_2 _Receptor Subtypes EP involved in AJC disassembly using butaprost, an EP2 specific receptor agonist and sulprostone and 17-phenyl trinor, both EP1 and EP3 agonist receptors and by TER measurements (Figure 5). Butaprost, sulprostone and 17-phenyl trinor were seen to cause a significant TER decrease after 15 and 30 min of treatment when compared to non-treated cells, however after 60 and 120 min there was a reverse effect on the TER measurements. It is important to emphasize that although sulprostone and 17-phenyl trinor PGE_2 _are EP1 and EP3 receptor agonists, in the concentration here used (1 μM), they have a higher affinity for the EP1 receptor [22-24]. This result was similar when the cells were treated with PGE_2 _or with its analogue, (16, 16-dm PGE_2_), which indicates the involvement of EP1 and EP2 receptors in a transient AJC disassembly mediated by PGE_2_.

### PGE_2 _module AJC disassembly through PKC signaling

**Figure 6:**
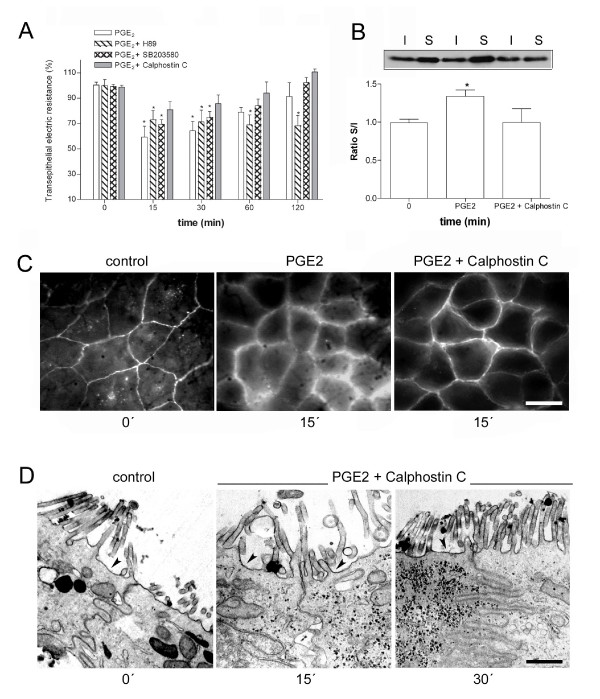
**PKC inhibition reverts the effect on the redistribution of Claudin-1 and the paracellular permeability caused by PGE_2_**. The TER was measured before and after treatment with 1 μM PGE_2 _and pretreated for 1 h with H-89, SB203580 and Calphostin C. Note that the decrease of the TER was PKC-dependent and H-89 abrogated the TER recovery at 60 and 120 min (A). Caco-2 cells were grown until confluence treated or pretreated with Calphostin C, prior to incubation with 1 μM PGE_2_and the redistribution of Claudin-1 was assessed by immunoblotting (B) and immunofluorescence (C). The effect of pretreatment with the inhibitor was also analyzed by using the ruthenium red technique and electron microscopy (D). Observe that PKC inhibition was able to block alterations caused by PGE_2_. Bars in B: 10 μm and in D: 1.2 μm.

There are no data about the cell-cell adhesion mechanisms mediated by PGE_2_, nor information concerning the signaling pathways involved in this event Thus, we decided to investigate downstream cell signaling mechanisms triggered by the EP receptors after PGE_2 _treatment (Figure 6). When cells were pretreated with SB203580, an inhibitor of p38 MAPK, it was possible to observe that the inhibitor did not prevent the drop of the PGE_2_- induced TER. Incubation of Caco-2 cells with H-89, a PKA blocker, did not prevent the TER decrease after 15 and 30 min of PGE_2 _treatment, but abolished the gradual TER re-stabilization promoted after 60 and 120 min. It is important to point out that, although the IC_50 _of H-89 for PKA is 48 or 135 nM [25], we used the concentration of 20 μM on the basis of previous studies showing that it is also able to inhibit PKA activity in culture cells [26,27]. Next, we verified if PKC is involved in this event and observed that pretreatment with Calphostin C, a well-known inhibitor of novel and conventional PKC isoforms, prevented the TER drops at all assessed times (Figure 6A). We further confirmed this later result by immunoblotting and immunofluorescence analysis using claudin-1, a TJ protein known to be involved in the regulation of the paracellular permeability [28]. Figures 6B and 6C show the reversible effect on the translocation to the TX-soluble fraction and redistribution of this protein through pretreatment with Calphostin C prior to incubation for 15 min with PGE_2_. In parallel, we also verified the effect of Calphostin C on the ultrastructural status of the AJC and TJ functionality using the ruthenium red marker. In Figure 6D it is possible to see that pretreatment with the PKC inhibitor completely blocked the permeation of the marker through the paracellular space caused after treatment for 15 and 30 min with PGE_2_. The ruthenium red in cells pretreated with Calphostin C was restricted at the apical region in a similar manner as in untreated cells. Taken together these results indicate that PKC is involved in the modulation of the AJC disassembly in PGE_2_- stimulated Caco-2 cells.

## Discussion

The loss of the AJC assembly by deleterious inflammatory mechanisms is an important problem in intestinal physiology due to the contribution of this structure to the maintenance of cell-cell adhesion. PGE_2 _has been implicated in essential physiological processes in the colon such as electrolyte transport, cell motility and in the pathogenesis of inflammatory bowel diseases where increased levels of PGE_2 _are observed in inflamed tissue [17]. Also, PGE_2 _has been reported as having a role in intestinal tumorigenesis [29]. Thus, there is strong evidence indicating a link between AJC regulation, intestinal inflammation and tumorigenesis. However the mechanisms underlying the PGE_2 _effects on AJC disassembly, a pivotal event at the beginning of the colorectal tumorigenesis, remain to be elucidated.

The results reported here show that PGE_2 _treatment caused transient differential redistribution of the AJC proteins in Caco-2 cells. We observed, by immunofluorescence, that AJ proteins, β-catenin and E-cadherin undergo significant alteration in localization. Similarly, TJ proteins claudin-1 and occludin showed an apparent redistribution from cell-cell contacts to the cytoplasm; however ZO-1 remained unaltered. By immunoblotting it was possible to observe a significant increase in the soluble TX-100 fraction of β-catenin, E-cadherin and claudin-1, but not for occludin. Furthermore, electron microscopy analysis of the subapical AJC region revealed wide spaces in this area in response to PGE_2_, but the TJ regions apparently remained unaltered. Concomitantly to these results, we showed a significant decrease of the TER, as well as increased permeation to ruthenium red marker in cells treated with PGE_2_. Since TJs are largely responsible for regulating paracellular permeability [30-32] it is probably that PGE_2 _acts directly against the components of these structures. The distribution analysis of claudin-1 tends to support this conclusion, since the translocation of this protein from the TX-100 insoluble fraction (cytoskeleton-linked proteins) to the TX-100 soluble fraction (cytoplasmic proteins) is associated with the drop of the TER and permeation to ruthenium red in response to PGE_2_. Several groups have described the involvement of pro-inflammatory cytokines, such as TNFα and interferon-γ, on AJC proteins and barrier function modulation [33,34]. In relation to PGE_2_, only one study carried out by Martin-Venegas et al., [35] reported increased paracellular permeability when differentiated Caco-2 cells were stimulated with PGE_2_. Nevertheless, in this study the AJC protein distribution was not carried out, different PGE_2 _concentrations were used and paracellular permeability alteration was only evidenced after 2 h of treatment. In our work here we also reported altered cytoskeleton-linked claudin-1, as evidenced by their translocation to TX-100 soluble fraction in PGE_2_-treated cells, corroborating with the paracellular permeability alteration. This result is consistent with studies showing that alterations in distribution or expression of claudin-1 play an important role in epithelial barrier function [36-38]. Adaptor ZO proteins have been documented as being responsible for the connection between claudins and the actin cytoskeleton [7]. The fact that we did not find distribution differences of ZO-1 in PGE_2- _treated cells, suggests that other adaptor proteins, not investigated here, could be mediating this linkage.

Prostanoids such as PGE_2 _exert their biological action through binding to four specific membrane receptors – the subtypes EP1 to EP4 that are G protein-coupled receptors [29]. The expression and involvement of these receptors in colorectal cancer has been reported [19,20,29]; however it is not known whether these receptors are involved in the regulation of the AJC disassembly and consequently contribute to carcinogenesis colorectal. Here, we showed the involvement of PGE_2 _receptor subtypes EP1 and EP2 in mediating AJC disassembly. This hypothesis is supported by the observation that 17-phenyl trinor PGE_2_, butaprost, and sulprostone caused a significant decrease in the TER at 15 and 30 min in a similar manner to PGE_2 _and its analogue. It has been reported that both, sulprostone and 17-phenyl trinor PGE_2 _are EP1 and EP3 receptor agonists, however in the concentration of 1 μM used in the present study, they have a higher affinity for the EP1 receptor [22,39,40]. Since previous results show that Caco-2 cells express only EP1 and EP2 subtypes of PGE_2 _receptors [41], we suggest that PGE_2 _mediates AJC disassembly through EP1 and EP2 receptors in this cell line.

It is known that EP2 receptors are coupled to PKA/adenyl cyclase and mediate the increase of intracellular cAMP [41] whereas ligand binding of EP1 is associated with phospholipase C and PKC activation [42]. On the other hand, studies have demonstrated the involvement of various cell signaling pathways such as: PKC, PKA, MAPK, and PI3K/Akt in the regulation of the TJ barrier function [7]. In our PGE_2 _stimulation model using Caco-2 cells, we found that PGE_2_-EP1 and -EP2 receptor signaling to decrease TER was predominantly linked to the PKC pathway, but not to PKA or p38MAPK. It is known that PKC has long been recognized to affect epithelial and endothelial barriers. This kinase consists of a family of Ser/Thr-specific kinases, which includes 12 known isozymes that can be classified into three subfamilies: conventional (α, β1, β2 and γ), novel (δ, ε, θ, ε and μ) and atypical (λ, τ and ζ), which differ in their mechanism of action, subcellular distribution, substrate type and expression [7]. In addition, several studies using different agents that perturb the epithelial junctional complex have demonstrated the involvement of various kinases in the phosphorylation and regulation of claudin proteins, however the mechanisms underlying this effect remain largely unknown. In relation to PKC, a recent study using three complementary molecular approaches and Caco-2 cells showed that the PKC-θ isoform plays various novel mechanisms in intestinal epithelium, namely: alterations of the claudin-1 and claudin-4 isotypes phosphorylation, membrane assembly, and distribution as well as permeability function in cell monolayer [38]. If this PKC isoform is responsible to mediate alteration in claudin-1 in Caco-2 cells treated with PGE2, remain to be elucidated. It is an important addition to studies on cell signaling mechanisms involving EP receptors in colorectal cancer that are usually aimed at analysing proliferation or apoptosis events, but not epithelial cell barrier function. For instance, it was reported that in an EP4 receptor expression model with HEK293 cells, cAMP signalling appears to play a minor role in proliferation [41]. By contrast, cAMP-dependent suppression of apoptosis by PGE_2 _seems to occur by a mechanism dependent on ERK and p38MAPK signaling, but not PKA [43]. Also, PGE_2_-dependent EGFR activation in human colorectal cancer cells appears to be variable, with responsive (LS-174) and unresponsive (DLD-1) cell lines described [16,44]. Recently, using a model of EP4 receptor overexpression in HT-29 cells, it was shown that PGE_2_-EP4 receptor signaling was linked predominantly to cAMP signaling and in low level to ERK activation, but not PKB/AKT signaling [20]. There is a clear significant heterogeneity of signaling pathways mediating PGE_2 _activities in different colorectal cells and the interplay between EP receptor subtypes, which are variably present on different colorectal cancer cell lines, may explain this event. Interestingly, we did not find PKA involvement in TER decrease, however it is recognized that cAMP can also signal in a PKA-independent manner via the cAMP-dependent guanine nucleotide exchange factor Epac1, which in turn activates Ras-GTPase Rap1 [45]. Additional studies are needed to elucidate if Epac 1 is involved in EP1 and EP2 receptor signaling pathways mediated by PGE_2_. Moreover, the fact that H-89 did prevented TER recovery after 60 and 120 min of treatment with PGE_2_, suggests that PKA activation is necessary for AJC restoration, which is consistent with data showing that PKA is related to positive regulation of cell-cell and cell-substrate adhesion [46].

In summary, we have shown that PGE_2 _can affect AJC architecture and function and that at least part of this effect is mediated through PKC activation in an event that requires the participation of EP1 and EP2 receptors and claudin-1 as an important target of PKC for the TJ-related effects.

## Conclusion

In this study we analyzed cell signaling mechanisms underlying PGE_2 _treatment on the Apical Junctional Complex assembly and function in a human colon cancer cell model. Using a physiologically relevant prostanoid dose it was possible to observe that AJC proteins are differentially redistributed and this effect was concomitant to an impairment of the paracellular permeability in Caco-2 cells. We demonstrated for the first time that PGE_2_-EP1 and EP2 receptor signaling regulates AJC disassembly through a mechanism that involves PKC, but not PKA or p38MAPK and reveals a critical role of claudin-1 in this event. Examination of these pathways can give a better understanding of the mechanisms concerning the loss of cell-cell adhesion and colon cancer progression and suggest new directions for potential therapy for this disease.

## Methods

### Antibodies and reagents

Rabbit polyclonal anti-claudin-1 (JAY.8), occludin and ZO-1 (Z-R1) antibodies were purchased from Zymed Laboratories, Inc. (San Francisco, CA, USA). Mouse monoclonal anti-E-cadherin (36) was purchased from BD Biosciences (San Diego, CA, USA). The rabbit polyclonal anti-beta-catenin was purchased from Sigma Chemical Co. (St Louis, MO, USA). The secondary antibodies Alexa 488-conjugated goat anti-rabbit IgG and Alexa 546-conjugated goat anti-mouse IgG were purchased from Molecular Probe (Eugene, OR). Peroxidase-conjugated goat anti-rabbit IgG were obtained from Zymed Laboratories, Inc. (San Francisco, CA, USA) and peroxidase-conjugated goat anti-mouse IgG from Sigma. Prostaglandin E_2_, butaprost, sulprostone, 16, 16-dm PGE_2 _and 17-phenyl trinor PGE_2 _were purchased from Cayman Chemical Company, (Ann Arbor, MI). SB203580, H-89 and Calphostin C were purchased from Biomol Res. Labs. Inc. (Plymouth Meeting, PA).

### Cell culture

Caco-2 cells (ATCC, # HTB-37, Rockville, MD, USA), a human colon cancer cell line were grown in Dulbecco Modified Eagle medium (DMEM) supplemented with 10% fetal bovine serum (FBS), penicillin G (60 mg/l) and streptomycin (100 mg/l) at 37°C in humidified atmosphere of 5% CO_2_/air. Culture medium was changed every 24 h to avoid nutrient depletion. All experiments were carried out when cells achieved confluence.

### PGE_2 _and EP2 agonist treatments

In order to determine the concentration of PGE_2 _in our experiments and on the basis of a kinetic study used by Pai et al [13], initially doses of 0.1 and 1 μM were tested. We determined that 1 μM was the concentration able to cause significant alterations on the AJC and was used in all subsequent experiments. Cell monolayers were serum-starved for 24 h then treated with 1 μM PGE_2 _for 15, 30 and 60 min. When indicated, cells were pre-treated with specific inhibitors of p38 MAPK (10 μM SB203580), PKA 20 μM (H-89) and PKC (500 nM Calphostin C), prior to PGE_2 _treatment.

The involvement of Prostaglandin E_2 _receptor EP subtypes was analyzed by using 1 μM butaprost, EP2 agonist receptor, and the EP1 and EP3 agonist receptors: 1 μM sulprostone and 1 μM 17 phenyl trinor PGE_2_. We used also 1 μM 16, 16-dimethyl Prostaglandin E_2 _(16, 16-dm PGE_2_), PGE_2 _analogous.

### Transepithelial Electrical Resistance (TER)

It is well known that TER is an instantaneous measurement that evaluates the degree of tightness and paracellular flux across epithelium [1]. In order to determine TJ functionality after PGE_2 _and EP agonist receptor treatments, we performed TER analysis at different times of treatments. Caco-2 cells were grown on Transwell polycarbonate filters 0.4 μm pore size (Costar, Cambridge, MA, USA) until confluent and treated as described above. TER values were determined using a Millicel-ERS system (Millipore Co, Billerica, MA, USA), with a 20A constant current. All TER values were normalized for the area of the filter (0.6 cm^2^) and were obtained after background subtraction (i.e., filter and bath solution). The results are expressed as percentage of total count (100%) values of each treatment in relation to the control group of three independent experiments.

### Immunofluorescence Microscopy

Cell monolayers were grown on sterile glass cover slips. After 15, 30 or 60 min of PGE2 treatment, cells were washed in PBS supplemented with 100 mM CaCl2 (PBS/CM), fixed and permeabilized with 100% methanol at -20°C for 20 min. Subsequently, they were re-hydrated in PBS/CM, incubated in blocking solution (0.2% BSA in PBS/CM) for 1 h and overnight at 4°C with primary antibodies anti-ZO-1 (1:25), anti-claudin-1 (1:25), anti-occludin (1:20), anti- β-catenin (1:2000) and anti-E-cadherin (1:100). Afterward they were incubated for 1 h at 37°C with the secondary antibodies Alexa 488-conjugated goat anti-rabbit IgG (1:500) or with Alexa 546-conjugated goat anti-mouse IgG (1:500). The cover slips were washed in PBS and mounted using n-propyl-gallate. Cell staining was detected using an Axiovert S 100 immunofluorescence microscope equipped with a CCD camera and KS 300 image analyzer (Carl Zeiss Inc., Jena, Germany).

### Transmission electron microscopy

Cells were cultured on Transwell polycarbonate filters, and after treatments they were washed in PBS and fixed in a solution containing 2.5% glutaraldehyde, 1% paraformaldehyde, 0.8% sucrose and 2 mM CaCl_2 _in 0.1 M cacodylate buffer, pH 7.4. Post-fixation was carried out in 1% osmium tetroxide (OsO_4_) in cacodylate buffer, containing 0.8% potassium ferrocyanide and 5 mM CaCl_2 _for 45 min. Subsequently, the cells were dehydrated with acetone and embedded in Epon resin. Ultrathin sections (60 nm) were obtained, stained with uranyl acetate and lead citrate and observed in a Zeiss CEM-900 transmission electron microscope (Carl Zeiss Inc., Jena, Germany).

In order to determine TJ functionality, cell monolayers were washed in PBS, and fixed for 60 min on the apical side with the solution above indicated containing 6 mg/ml of ruthenium red. Cells were washed three times with cacodylate buffer containing ruthenium red for 10 min each and post fixed with 1% OsO4 and 6 mg/ml ruthenium red in cacodylate buffer for 45 min. Subsequently, they were dehydrated in acetone series and embedded in Epon resin. Ultrathin sections were obtained, stained for 3 min with lead citrate only and observed in a Zeiss CEM-900 transmission electron microscope (Carl Zeiss Inc.).

### Cell extraction in Triton X-100 and immunoblotting

Samples were rinsed three times in PBS/CM and incubated for 20 min at 4°C in extraction buffer CSK: 50 mM NaCl, 10 mM piperazine-1, 4-bis (2-ethanesulfonic acid) (Pipes), pH 6.8, 3 mM MgCl_2_, 0.5% TritonX-100, 300 mM sucrose, 1 mM orthovanadate, 20 mM NaF, and protease inhibitor cocktail (1:100, Sigma Chemical Co.) for 20 min at 4°C. Cells were scratched from plates, homogenized and centrifuged at 10,000 g for 10 min at 4°C. The supernatant corresponding to the TX-100 soluble fraction (cytosolic proteins) was removed and stored at -20°C. The pellet was resuspended in SDS buffer: 20 mM Tris-HCl, pH 7.5, 5 mM ethylenediamine-tetraacetic acid (EDTA), 2.5 mM [ethylenebis(oxyethylenenitrilo)] tetra (EGTA), 1% sodium dodecyl sulfate (SDS) and boiled at 100°C for 10 min. After centrifugation for 10 min at 10,000 g the supernatant, corresponding to the TX-100 insoluble fraction (cytoskeleton-linked proteins), was gently removed and stored at -20°C.

Equal amounts of protein (30 μg), of cell fractions were electrophoretically separated by SDS-PAGE in 7.5% or 12% gels and transferred to nitrocellulose sheets using a semidry transfer cell (BioRad, Hercules, CA, USA) at 10 V for 60 min. [47]. Then, the membranes were blocked for 1 h with TBS-T: 20 mM Tris-HCl, pH 7.6, 137 mM NaCl and 0.1% v/v Tween 20, containing 5% low-fat dried milk and incubated overnight with primary antibodies: anti-occludin (1:250), anti-E-cadherin (1: 2,000), anti-claudin-1 (1:250) and anti-ZO-1 (1:250). After washing, membranes were incubated for 1 h with peroxidase-conjugated goat anti-rabbit IgG (1:10,000) or peroxidase-conjugated goat anti-mouse IgG (1:40,000). Proteins were visualized using an enhanced chemiluminescence kit (Amersham Pharmacia Biotech, Buckingham, UK). Band images were quantified by optical density using the LabWorks 4.6 software (BIO RAD, Upland, CA).

### Statistical analysis

Transepithelial Electric Resistance data were normalized to percentage and analyzed by one-way ANOVA followed by Bonferroni posttest for comparison between groups using GraphPad Prism version 4.0 for Windows (GraphPad Software, San Diego, CA). Densitometric analyses, which are comparisons between non-treated (which was normalized to 1) and treated samples, were carried out using Student's *t*-test. All values in text and figures are means ± S.E.M of three independents experiments. Significantly different: * (P < 0.05).

## Authors' contributions

Author JAMD conceived of the study. MNT and JAMD designed and carried out all the experiments reported in the manuscript. BLD and WS participated in the design and analysis of PGE_2 _and ultrastructural experiments, respectively. JAMD participated in the coordination of the study and drafted the manuscript with input from all authors. All authors read and approved the final manuscript.
